# Weighting sequence variants based on their annotation increases the power of genome-wide association studies in dairy cattle

**DOI:** 10.1186/s12711-019-0463-9

**Published:** 2019-05-10

**Authors:** Zexi Cai, Bernt Guldbrandtsen, Mogens Sandø Lund, Goutam Sahana

**Affiliations:** Department of Molecular Biology and Genetics, Center for Quantitative Genetics and Genomics, Aarhus University, 8830 Tjele, Denmark

## Abstract

**Background:**

Genome-wide association studies (GWAS) are widely used to identify regions of the genome that harbor genetic determinants of quantitative traits. However, the multiple-testing burden from scanning tens of millions of whole-genome sequence variants reduces the power to identify associated variants, especially if sample size is limited. In addition, factors such as inaccuracy of imputation, complex linkage disequilibrium structures, and multiple closely-located causal variants may result in an identified causative mutation not being the most significant single nucleotide polymorphism in a particular genomic region. Therefore, the use of information from different sources, particularly variant annotations, was proposed to enhance the fine-mapping of causal variants. Here, we tested whether applying significance thresholds based on variant annotation categories increases the power of GWAS compared with a flat Bonferroni multiple-testing correction.

**Results:**

Whole-genome sequence variants in dairy cattle were categorized according to type and predicted impact. Then, GWAS between markers and 17 quantitative traits were analyzed for enrichment for association of each annotation category. By using annotation categories that were determined with the variants effect predictor software and datasets indicating regions of open chromatin, “low impact” variants were found to be highly enriched. Moreover, when the variants annotated as “modifier” and not located at open chromatin regions were further classified into different types of potential regulatory elements, the high impact variants, moderate impact variants, variants located in the 3′ and 5′ untranslated regions, and variants located in potential non-coding RNA regions exhibited relatively more enrichment. In contrast, a similar study on human GWAS data reported that enrichment of association signals was highest with high impact variants. We observed an increase in power when these variant category-based significance thresholds were applied for GWAS results on stature in Nordic Holstein cattle, as more candidate genes from previous large GWAS meta-analysis for cattle stature were confirmed.

**Conclusions:**

Use of variant category-based genome-wide significance thresholds can marginally increase the power to detect the candidate genes in cattle. With the continued improvements in annotation of the bovine genome, we anticipate that the growing usefulness of variant category-based significance thresholds will be demonstrated.

**Electronic supplementary material:**

The online version of this article (10.1186/s12711-019-0463-9) contains supplementary material, which is available to authorized users.

## Background

Cattle is one of the most important domestic animals in human history. Both breeding programs and genetic studies in cattle depend largely on the availability of a reliable cattle reference genome [1] and reference populations [2]. In addition, genome-wide association studies (GWAS) have identified valuable links between genetic variants and variations in complex traits [3, 4]. For example, numerous GWAS have been conducted in cattle to investigate production traits such as milk yield [5, 6], milk composition [7], and mastitis [8–10]. However, GWAS alone cannot distinguish causative variants from variants which are in perfect, or near-perfect, linkage disequilibrium (LD) with them.

To address this problem, additional information from independent sources are needed. For example, gene expression data have facilitated the identification of candidate genes from GWAS data [11], and expression quantitative trait loci (eQTL) data [12, 13] have helped map causative variants within regulatory regions. More recently, Brown et al. [14] proposed a ‘causal-variant evidence mapping using nonparametric resampling’ (CaVEMaN) method to pinpoint causative mutations in eQTL studies. By integrating eQTL data with results from GWAS, genes with expression levels that are associated with complex traits due to pleiotropic effects (e.g., when both gene expression and trait variation are affected) can be identified [13]. However, large-scale eQTL studies can be expensive because they require generation of RNAseq data specific to the population under study, especially in the case of livestock species for which initiatives such as the GTEx [15] project in humans do not exist.

Due to many reasons, such as LD, inaccuracy of imputation, random sampling errors, etc., the lead single nucleotide polymorphism (SNP) may not be the causative one [6]. Using additional information to prioritize variants within the QTL interval has become a popular strategy [16]. It was recently demonstrated that the use of a variant annotation tool [17] and its evolutionary conservation score [16] can help prioritize variants. In particular, variant annotation can be used across different studies without being tissue- or trait-specific. The power to identify associations between genetic variants and phenotypes may be further improved by using functional annotation information [18–20]. For example, Sveinbjornsson et al. [21] reported an increase in power for the detection of associations when an annotation enrichment-based weighted Bonferroni adjustment was used to correct for family-wise error rate (FWER).

In this study, we implemented a previously proposed, category-based Bonferroni adjustment based on the enrichment (the probability of a causal variant being from a category divided by the probability of a non-causal variant being from the same category) of variant annotations observed for association signals [21] that were obtained from a GWAS conducted in Nordic Holstein cattle. This adjustment is based on the hypothesis that different types of variants have varying probabilities of being causal mutations, which means the enriched categories of variants could have lower thresholds estimated from their enrichment. The GWAS results for 17 quantitative traits were used to extract the lead SNP along with other significant SNPs showing LD (r^2^ > 0.2) with the lead SNPs, as potential causal variants to estimate the enrichment of each of the annotated variants’ categories. We make the hypothesis that the category-based significance threshold will increase the power of a GWAS study. We tested this hypothesis by performing an association study on stature in cattle and comparing the results with those of a previously reported large meta-analysis in cattle stature and genes reported for human height.

## Methods

### Phenotype and genotype data

Since, no animal experiments were performed in this study, approval from an ethics committee was not required.

Phenotypic records on 17 traits/indices for Nordic Holstein cattle were obtained from a central national database (Nordic Cattle Genetic Evaluation (NAV), http://www.nordicebv.info/). For details on the genetic evaluations performed for these 17 traits/indices in Nordic countries, see http://www.nordicebv.info/production. The phenotypic values used in the association analysis included de-regressed proofs that were derived for animals based on the effective daughter contributions of sires and maternal grandsires [22, 23], which were obtained from the NAV routine genetic evaluations by using the MiX99 software [24]. De-regressed proofs were available for 5373 sires (the total number of animals varying according to trait). A short description of the 17 traits/indices is presented in Additional file 1: Table S1.

An association study was performed by using imputed WGS data, as previously described by Iso-Touru et al. [5] and Wu et al. [25]. A total of 4921 bulls were genotyped with versions 1 or 2 of the Illumina BovineSNP50 BeadChip (54 k) system (Illumina, San Diego, CA, USA). The 54 k genotypes were imputed to the WGS level by using a 2-step approach [26]. First, all the animals were imputed to a high-density (HD) level, by using IMPUTE2 v2.3.1 and a multi-breed reference of 3383 animals (1222 Holsteins, 1326 Nordic Red Dairy Cattle, and 835 Danish Jerseys), which had previously been genotyped with the Illumina Bovine HD BeadChip [27]. The distribution of imputation accuracies according to minor allele frequency is described in [25]. These imputed HD genotypes were imputed with Minimac2 [28] to the WGS level by using a multi-breed reference of a total of 1228 animals that included 1148 animals from *Run4* of the 1000 Bull Genomes Project [2] (288 Holstein, 56 Nordic Red Dairy cattle, 61 Jersey cattle, and 743 cattle from other breeds [2]), and 80 animals from Aarhus University (23 Holsteins, 30 Nordic Red Dairy cattle, and 27 Danish Jerseys). The 1000 Bull Genome Project data are described in Daetwyler et al. [2] and the whole-genome sequence data from Aarhus University are described in Brøndum et al. [29]. A total of 22,751,039 bi-allelic variants were present in these imputed sequence data. After excluding SNPs with a minor allele frequency lower than 1%, and SNPs that deviate from Hardy–Weinberg proportions (P < 1.0^−6^), 16,503,508 SNPs on 29 autosomes in Nordic Holstein cattle were retained for association analyses.

### Methodology for the detection of multiple QTL

We performed a GWAS according to a previously described approach [6]. First, a single SNP GWAS analysis was performed by using GCTA [30] for each chromosome as the first round. Next, SNPs were ranked based on their − log_10_ (P) values. The SNP with the highest − log_10_ (P) value, referred to as the lead SNP, was identified for each chromosome. If the − log_10_ (P) value of the lead SNP exceeded 8.5 (a threshold value representing a 0.05 type I error-rate after Bonferroni correction for 16,503,508 simultaneous tests, e.g., − log_10_ (P) ≈ 8.5), the SNP genotype dosage was fitted as a covariate, and rerun in association analyses for the same chromosome as a second round. If the result of this second round detected another SNP with a − log_10_ (P) value exceeding 8.5, and this SNP was also significant in the first round (e.g., − log_10_ (P) > 8.5), we fitted it as another covariate, and then scanned the chromosome in a third round. This same procedure was repeated for each chromosome until no additional SNPs remained significant. A list of the lead SNPs identified in each round was compiled. In each round, we checked whether the lead SNP was the only significant SNP identified within ± 1 Mb of flanking region. If it was, the SNP was not considered as a lead SNP since it could represent a false positive or be mapped to a wrong location in the genome. Details regarding the 17 traits and the GWAS results are in Additional file 1: Table S1.

### GWAS for stature in Nordic Holstein cattle

Stature in cattle is measured from the top of the spine between the hips to the ground. In Denmark, this trait is measured in cm. We performed a GWAS for stature according to the method described above. However, first we removed extreme phenotypic records according to Tukey’s rules of quartiles ± 1.5 × interquartile range. The remaining 4832 phenotypic records were associated with 15,535,049 imputed SNPs. The number of markers used for association with stature differs from that used in the GWAS conducted for the 17 traits (described above) since the set of sires was not exactly the same in both analyses.

### LD estimation and variant annotation

PLINK was used to estimate pairwise LD (r^2^) between lead SNPs and all the other SNPs on the same chromosome. All SNPs that had an r^2^ with the lead SNPs higher than 0.2 were extracted. The SNPs that were not significant in the association study were discarded in order to generate a list of possible causal variants. These SNPs were annotated with the variants effect predictor (VEP) (version 92.0) software [17]. The variants were subsequently classified into annotation categories according to the impact for the consequence type predicted by VEP. When a SNP had multiple annotations, the annotation with the highest impact predicted by VEP was retained. Information on transposase-accessible chromatin, i.e. ATAC-seq peaks [31], as well as histone modifications, i.e. H3K27Ac and H3K4me3 peaks [32], were retrieved from previously published studies. The locations of the UTR regions were obtained from Ensembl [33]. The locations of predicted regulatory elements (RE) were also obtained from a previous study [34], while the locations of non-coding RNAs (ncRNAs) were retrieved from the RNAcentral database [35].

### Assessment of category enrichment and category-based Bonferroni correction

Methods to assess the enrichment of each category, the enrichment confidence intervals, and weighted Bonferroni corrections were previously described [21]. We classified all the variants based on the VEP annotation: (1) high impact variants (e.g., stop_gained, stop_lost, start_lost, frameshift, splice_acceptor, and splice_donor variants), (2) moderate impact variants (missense_variants), (3) low impact variants (synonymous, stop_retained, upstream_gene, downstream_gene and splice_region variants), and (4) other variants (including SNPs with a consequence predicted as “modifier”). In addition, we further classified other variants (annotated by VEP as “modifier”) using open chromatin (OC) information to two categories, which resulted in a total of five categories: (1) high impact variants, (2) moderate impact variants, (3) low impact variants, (4.1) OC variants (annotated by VEP as “modifer” and located at ATAC-seq peaks [31] or H3K27Ac and H3K4me3 peaks [32]), and (4.2) variants with no known function i.e. NKF variants (including SNPs with a consequence predicted as “modifier”, which were not located in OC). Finally, we further classified the NKF variants (category-4.2 above) into four categories leading to a total of eight categories. The four NKF categories were: (4.2.1) variants located within 5′ and 3′ untranslated regions (UTR), (4.2.2) variants located in predicted RE according to a recently proposed algorithm based on conservation among mammals [34], (4.2.3) variants located within ncRNAs retrieved from the RNAcentral database [35], and (4.2.4) variants with no known information (NKI) predicted as “modifier” and not located in any of these first three types of sequence. First, we considered UTR, since these regions mediate the initiation and termination of translation. Next, we considered the experimental datasets of accessible chromatin (ATAC-seq) [31] and active motifs (H3K27Ac and H3K4me3) [32]. Second, we considered RE for two reasons: (1) because promoters and transcription factor binding sites are near transcription start sites [36, 37], and regions proximal to genes tend to exhibit greater enrichment of significantly associated variants in GWAS [38]; and (2) predicted RE can potentially help identify causal mutations [34]. ncRNAs play a major role in gene expression regulation [39], although their specific functions are largely unknown [40]. The detailed classification of variants is in Additional file 1: Table S2.

According to Sveinbjornsson et al. [21], we estimated the probability of a causal variant being a particular annotation type (according to the five or eight categories that we established in this study) by using a maximum likelihood method. Accordingly, the enrichment of an annotation category was estimated based on the probability of a causal variant being from a class, divided by its genomic frequency. The significance threshold for each annotation category was then estimated based on a category-based Bonferroni correction threshold that was established based on enrichment of the annotation class. For example, for a total number of sequence variants tested (*T*), the number of variants in an annotation category *C* (*T*_*C*_), and enrichment of category *C* (*e*_*C*_), *c*_*j*_ is the category to which the *j*th sequence variant belongs with enrichment *e*_*Cj*_, and the weight for the *j*th sequence variant is [21]:

$$w_{j} = \frac{{e_{cj} }}{{\frac{1}{T}\mathop \sum \nolimits_{C} T_{c} e_{c} }},$$

$$P_{{wt_{j} }} = P_{bc} \times w_{j} ,$$

where $P_{{wt_{j} }}$ and *P*_*bc*_ are weighted significance thresholds for the *jth* variant and Bonferroni corrected FWER, respectively.

### Bootstrapping and confidence interval estimation for enrichment

Association signals (QTL) were resampled 100 times with replacement. For each resample, we estimated enrichment for annotation categories and calculated the averages and 95% confidence intervals. The code for bootstrapping procedure is included in the R code provided in Additional file 2 together with the code for enrichment estimation.

## Results

### GWAS for 17 traits in Nordic Holstein cattle

A total of 5373 animals with 16,503,508 imputed SNPs were subjected to GWAS for 17 traits. A previously described pipeline [6] was used to detect the ‘lead’ variants that showed the highest association for each association signal. A total of 261 QTL (see Additional file 1: Table S1) were detected with a genome-wide association significance threshold of − log_10_(*p*) > 8.5. Significant associations were observed for 16 of the 17 traits examined (see Additional file 1: Table S1). Due to long-range LD in the bovine genome [5], sequence variants that were in LD with the lead SNPs (r^2^ > 0.2) and genome-wide significant were identified as possibly causal. In total, 78,593 possibly causal variants on 29 autosomes were selected for further analysis.

### Annotations for all possible causal variants

**Fig. 1:**
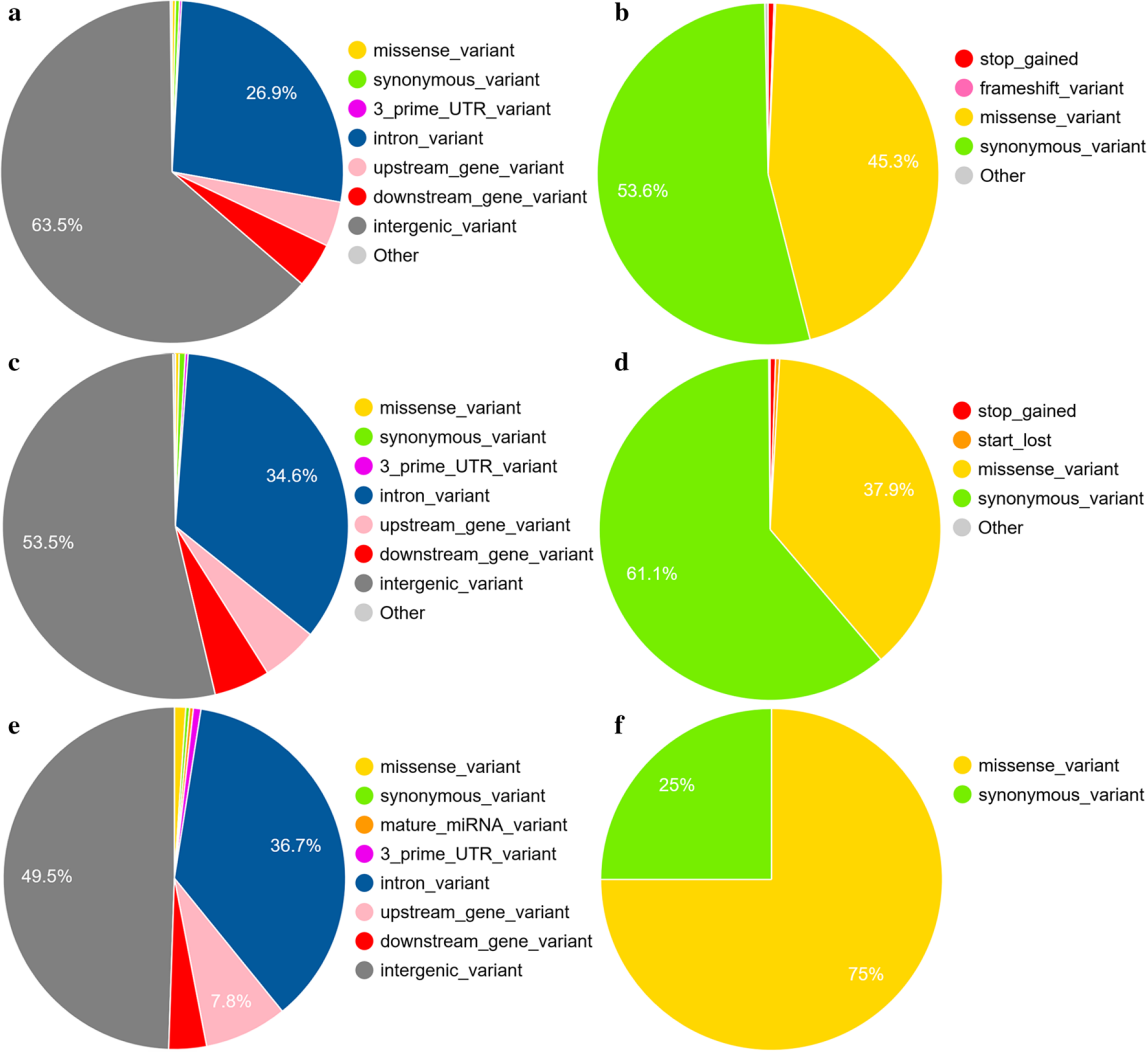
VEP annotations for the variants examined in this study. **a** Overall distribution of VEP annotations for all variants. **b** Annotation distribution for the variants present in coding sequences of all variants (**c**), for possible causal variants from the LD analysis, **d** for possible causal variants in coding sequences from the LD analysis, **e** for lead variants, and **f** for lead variants in coding sequences

The variant effect predictor (VEP) software (version 92.0) [17] was used to predict the maximal consequence of the variants on the nearest genes (e.g., within 5-kb flanking regions). A summary of the annotations obtained is in Fig. 1. Most of the annotated variants are intergenic variants or intron variants (Fig. 1a). Among the coding sequences, the most abundant variants are synonymous variants and missense variants (Fig. 1b). We also examined the distribution of annotations among the possible causal variants and the lead variants. The total number of variants in these two sets were equal to 78,593 and 261, respectively. The overall distributions of the annotations for these two groups (Fig. 1c–f, respectively) were similar to that of the entire set of variants (Fig. 1a, b). We also observed that no high impact variants (e.g., “stop gained” or “start lost” variants) were present among the lead SNPs identified.

### Enriched annotations and annotation-based significance thresholds based on VEP annotation

**Fig. 2:**
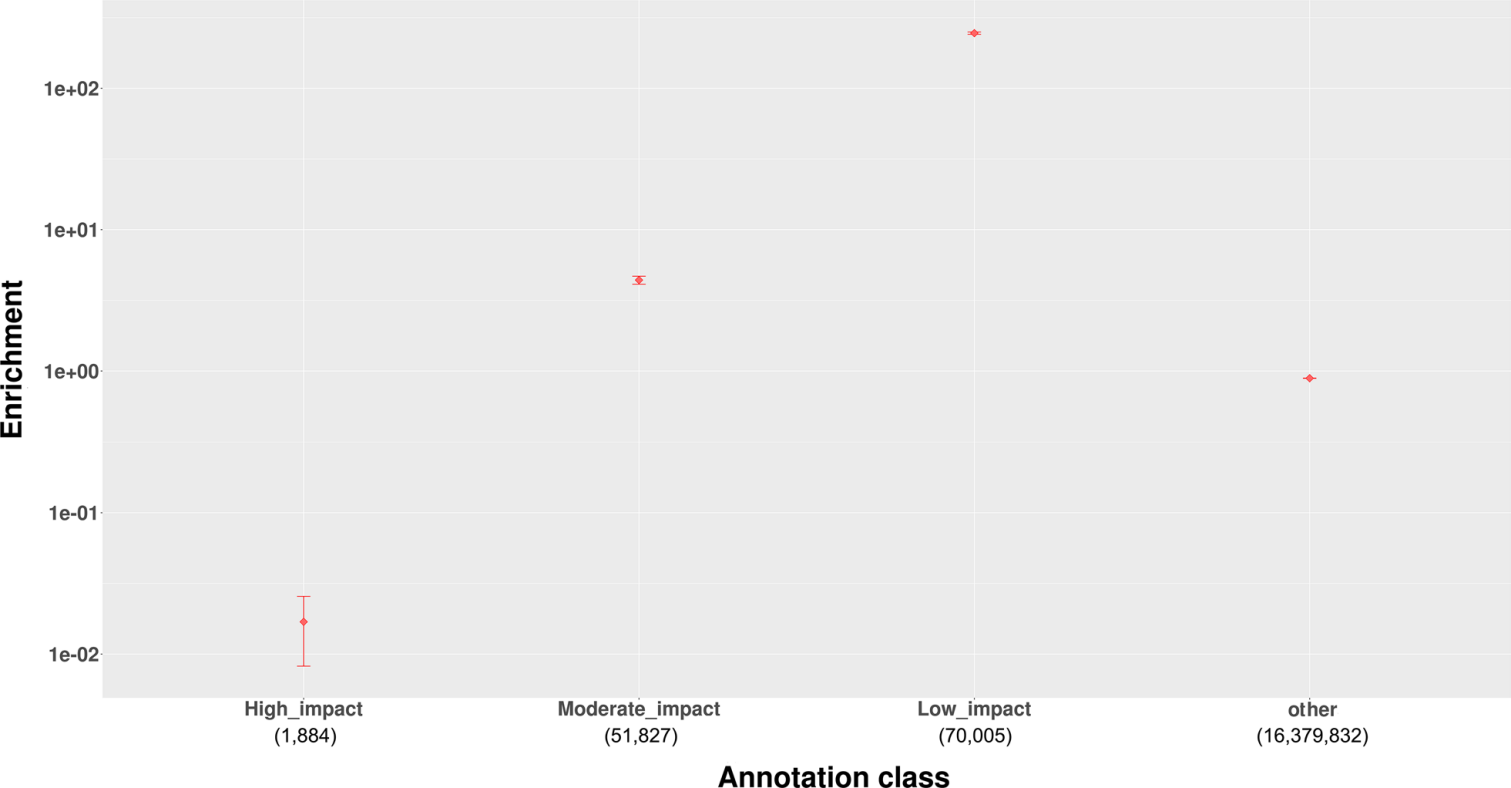
Enrichment of VEP and OC annotations for the four-category annotation system. Relative enrichments for four categories of variants. The error bars indicate the standard errors derived by bootstrapping. Enrichment is shown on the y-axis on a logarithmic scale

Based on the VEP-derived annotations, we classified all the annotation types obtained into four categories: (1) high impact variants, (2) moderate impact variants, (3) low impact variants, and (4) other variants. The other variants included SNPs that were annotated by VEP as intergenic variants, and those with a consequence predicted as “modifier”. A plot of category enrichment is in Fig. 2. In contrast with the results of previous GWAS that involved quantitative and binary phenotypes in humans [21], low impact variants were the most enriched (245-fold enrichment) category instead of the high impact variants (Table 1). The next most enriched category was the moderate impact variants, and these exhibited a fivefold enrichment (Table 1). We did not observe enrichment for high impact variants and ‘other variants’.

**Table 1:** Enrichment of four annotation categories and their category-based significance thresholds

Category	Number of possible causal variants	Enrichment	Confidence interval	Category-based significance threshold
High impact	19	0.017	− 0.00013 to 0.034	NA*
Moderate impact	391	4.40	3.84–4.96	6.88e−9
Low impact	799	245.45	236.44–254.45	3.84e−7
Other	102,214	0.89	0.88–0.89	NA*

The confidence interval for each degree of enrichment is the 95% confidence interval obtained from bootstrapping resampled QTL 100 times

*Indicates that the category-based significance was not calculated for this annotation class since there was no enrichment for this category

### Incorporation of information of open chromatin

**Fig. 3:**
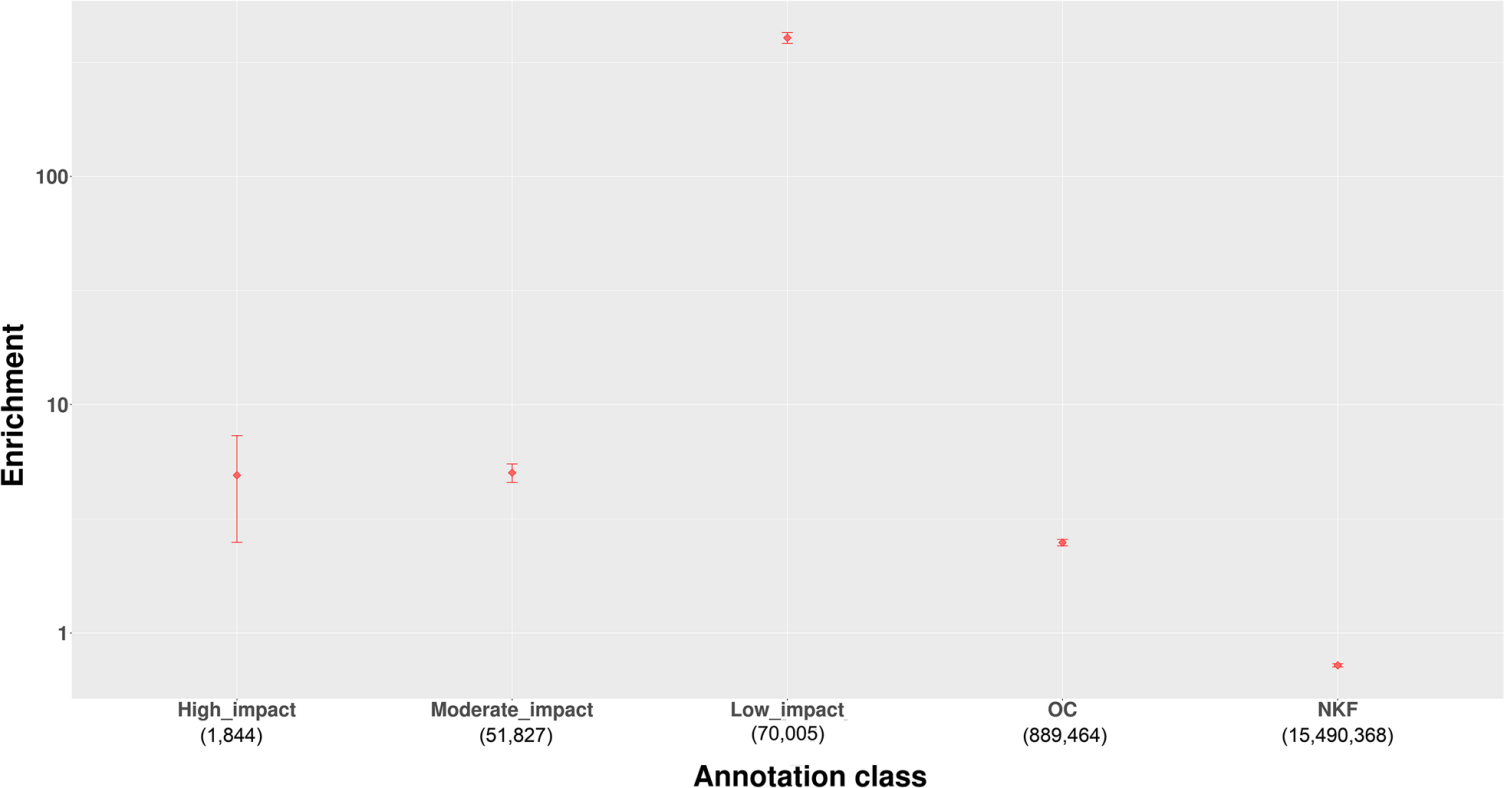
Enrichment of VEP and OC annotations for the five-category annotation system. Relative enrichments for four categories of variants. The error bars indicate the standard errors derived by bootstrapping. Enrichment is shown on the y-axis on a logarithmic scale

While an extensive dataset of DNase I hypersensitivity sites (DHS) is available for the human genome [21], such data are much more limited for the bovine genome. However, an assay for transposase-accessible chromatin with a high-throughput sequencing (ATAC-seq) dataset for cattle was recently generated to explore accessible chromatin regions in the bovine genome [31]. In addition, a histone modification dataset (for H3K27Ac and H3K4me3) was created to mark active motifs across the bovine genome [32]. Therefore, in combination with VEP-derived annotations, we classified all the annotation types obtained into five categories: (1) high impact variants, (2) moderate impact variants, (3) low impact variants, (4) open chromatin (OC) variants, and (5) variants with no known function (NKF). The latter included variants that are not known to affect biological processes, including SNPs annotated by VEP as intergenic variants, and those with a consequence predicted as “modifier” which were not located within OC regions. A plot of category enrichment is in Fig. 3. In contrast with the results of a previous GWAS that involved quantitative and binary phenotypes in humans [21], low impact variants were the most enriched (405-fold enrichment) category instead of the high impact variants. Moreover, in spite of a large variance in the interval of enrichment for the low impact variants, the lower boundary still represented a high level of enrichment (Table 2). The next most enriched category was the moderate impact variants, and these exhibited a fivefold enrichment, followed by the high impact variants that exhibited a fourfold enrichment. The lower boundary of the high impact variants was not enriched and this category included only 19 variants (Table 2). Furthermore, enrichment was observed only for 2 of 100 replicates (with values of 177-fold and 133-fold, respectively; (see Additional file 3: Table S3). Therefore, we did not consider that these high impact variants were true positives. Finally, the OC category exhibited a 2.5-fold enrichment. Based on these enrichment values, a category-based significance threshold was calculated for each variant category (Table 2).

**Table 2:** Enrichment of five annotation categories and their category-based significance thresholds

Category	Number of possible causal variants	Enrichment	Confidence interval	Category-based significance threshold
High impact	19	4.92	0.18–9.65	5.87e−9
Moderate impact	391	5.04	4.13–5.96	6.00e−9
Low impact	799	405.08	362.29–447.88	4.82e−7
Open chromatin	7227	2.49	2.33–2.65	2.96e−9
No known function	94,987	0.72	0.70–0.74	NA*

The confidence interval for each degree of enrichment is the 95% confidence interval obtained from bootstrapping resampled QTL 100 times

*Indicates that the category-based significance was not calculated for this annotation class since there was no enrichment for this category

### Incorporation of additional genomic information to address NKF variants

**Fig. 4:**
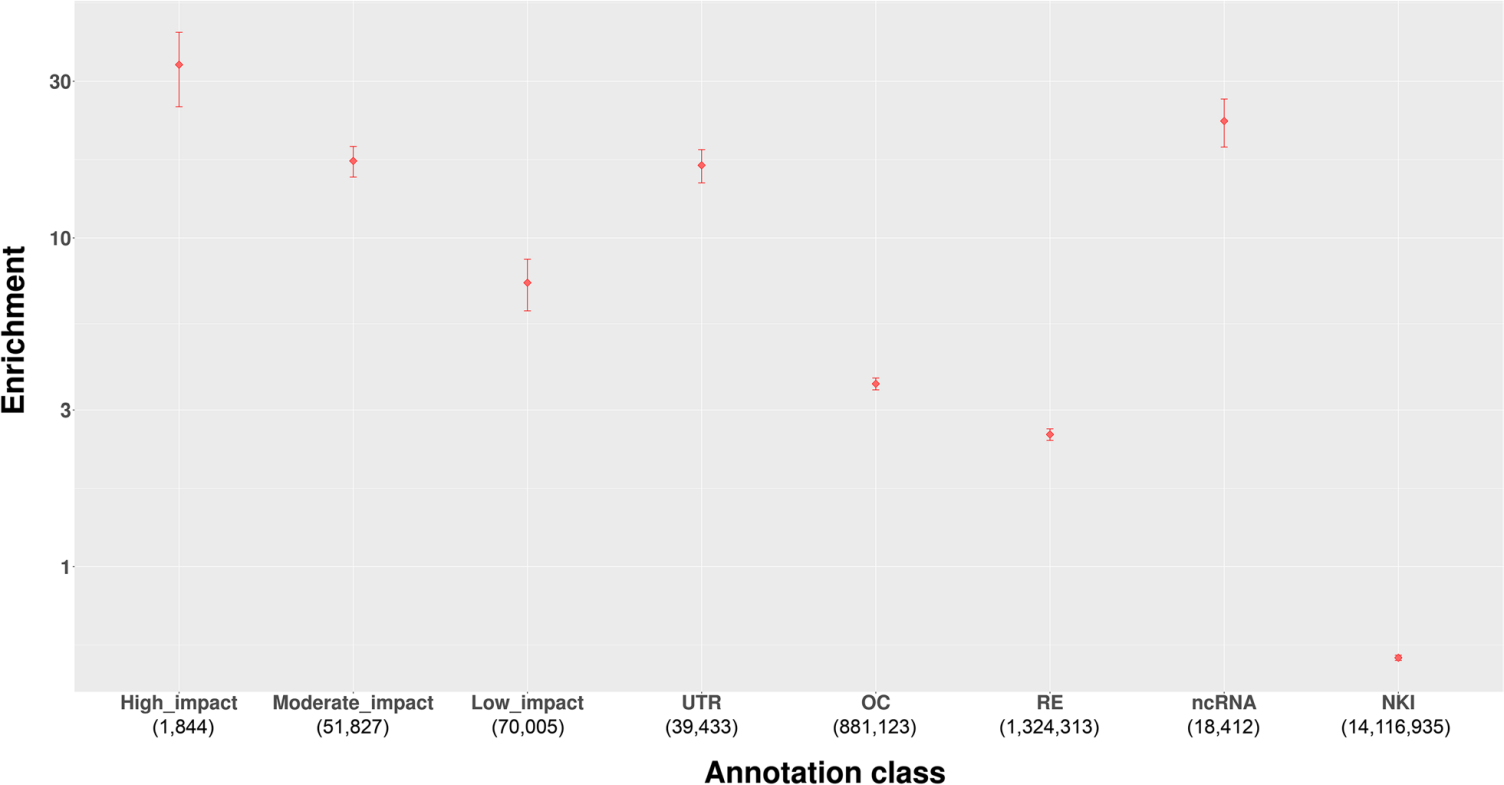
Enrichment of VEP annotations and further classification of NKF variants into UTR, RE, and ncRNA in an eight-category annotation system. Relative enrichments for seven categories of variants are presented. Error bars indicate standard error values derived from bootstrapping. Enrichment is shown on the y-axis according to a logarithmic scale

The enrichment observed for each of these eight categories (including sub-categories from ‘modifiers’) is provided in Table 3 and represented in Fig. 4. The most enriched variants were high impact variants (33.69-fold enrichment), which is similar to the enrichment profile of human variants reported in [21]. The moderate impact variants exhibited a 17.16-fold enrichment and the low impact variants exhibited a 7.30-fold enrichment. The ncRNA category exhibited a 22.70-fold enrichment, the UTR category exhibited a 16.64-fold enrichment, and the OC and RE categories exhibited 3.59-fold and 2.53-fold enrichments, respectively.

**Table 3:** Enrichment of eight variant categories and their category-based significance thresholds

Category	Number of possible causal variants	Enrichment	Range of enrichment	Significance threshold
High impact	19	33.69	16.85–50.54	1.02e−7
Moderate impact	391	17.16	13.56–20.75	5.20e−8
Low impact	799	7.30	4.74–9.87	2.21e−8
3′ and 5′ UTR	343	16.64	12.56–20.43	5.04e−8
Open chromatin	7152	3.59	3.30–3.89	1.09e−8
Regulatory elements	9520	2.53	2.33–2.73	7.65e−9
Non-coding RNAs	95	22.70	15.27–30.12	6.88e−8
No known information	85,104	0.53	0.51–0.55	NA*

The confidence interval for each degree of enrichment is the 95% confidence interval obtained from bootstrapping resampled QTL 100 times

*Indicates that the category-based significance was not calculated for this annotation class since there was no enrichment for this category

### Variant annotation-based significance thresholds in GWAS

To assess the power of using annotation category-based significance thresholds, we applied this approach to a GWAS conducted for stature in cattle and identified 35 QTL on 21 chromosomes (see Additional file 4: Figure S1 and Additional file 3: Table S4). The number of significant variants within each of the four, five and eight categories of classified annotations (as described above) are in Tables 4, 5 and 6, respectively. When adjusted thresholds based on the four-category classifications were used in comparison with a flat Bonferroni multiple-testing correction across the tested variants, the total number of significant variants increased from 58,539 to 58,992 (Table 4). Then, when we checked whether the additional genes that were identified with the category-based thresholds had been previously identified as candidate genes in a meta-analysis study on bovine stature [41], we found that *TNNI2* and *TCP11* were identified by the new significant variants (n = 453). Subsequently, when we checked for overlap between the newly identified genes and those from a previous study on human height [42], we detected five overlapping genes i.e. *ANKRD52*, *DNMT1*, *SCN4A*, *TP53I13*, and *TCP11* that may be associated with cattle stature and human height.

**Table 4:** Comparison of the numbers of significantly associated SNPs identified by applying an annotation category-based Bonferroni correction to each annotation category

Category	Number of significant variants	
	With conventional Bonferroni correction	With category-based Bonferroni correction
High impact	9	9
Moderate impact	208	234
Low impact	378	805
Other	57,944	57,944
Total	58,539	58,992

**Table 5:** Comparison of the numbers of significantly associated SNPs identified by applying an annotation category-based Bonferroni correction to each annotation category

Category	Number of significant variants	
	With conventional Bonferroni correction	With category-based Bonferroni correction
High impact	9	10
Moderate impact	208	226
Low impact	378	852
Open chromatin	3700	3661
No known function	54,244	54,244
Total	58,539	58,993

**Table 6:** Numbers of significantly associated SNPs identified by using different significance thresholds for each annotation category

Category	Number of significant variants	
	Bonferroni correction	Category-based Bonferroni correction
High impact	9	11
Moderate impact	208	334
Low impact	378	507
3′ and 5′ UTR	194	296
Open chromatin	3657	4620
Regulatory elements	6621	7911
Non-coding RNAs	69	109
No known function	47,403	47,403
Total	58,539	61,191

When adjusted thresholds based on the five-category classifications were used versus a flat Bonferroni multiple-testing correction across the tested variants, the total number of significant variants increased from 58,539 to 58,993 (Table 5). The list of identified genes is similar to that obtained with the four-category classification threshold, except that one additional gene *FBP1* was included. When adjusted thresholds based on the eight-category classifications were used versus a flat Bonferroni multiple-testing correction across the tested variants, the total number of significant variants increased from 58,539 to 61,191 (Table 6) and the newly identified variants (n = 2652) included *TNNI2* and *TCP11*, as obtained by using the four- or five-category classification thresholds. When we checked for overlap between these newly identified genes with the previous study on human height [42], in this case, we detected more genes i.e. *GHR*, *THADA*, *RPS6KA1*, *TP53I13*, *TCP11*, *VGLL4*, *KCNJ12*, *PPP2R3A*, *GCKR*, and *ZBTB38* that may be potentially relevant for cattle stature and human height.

## Discussion

The goal of this study was to investigate whether categories of variants with different probabilities of being functional can be identified based on enrichment values obtained from categorization of GWAS results. When we classified all the variants on the basis of VEP annotation only, we observed a greater enrichment of “low impact” variants and a limited enrichment of “moderate impact” variants (Table 1). When we classified all the variants into five categories based on: (1) their impact predicted by VEP, (2) an ATAC-seq dataset indicating accessible chromatin regions [31], and (3) a histone modification dataset (involving H3K27Ac and H3K4me3) [32], we observed a greater enrichment of “low impact” variants and limited enrichment of “high impact”, “moderate impact”, and OC variants (Table 2). In contrast, when we classified the original GWAS variants into eight categories, a more than tenfold enrichment was observed for the “high impact”, “moderate impact”, UTR, and ncRNA categories (Table 3), whereas “low impact” variants, OC, and RE categories exhibited a limited enrichment. This variant enrichment profile differs from that reported in a study on human data [21], for which the enrichments observed were in line with the magnitude of the predicted consequences of the variants. Thus, the variants with consequences that were predicted to be more severe displayed a greater enrichment. One reason for this difference in results may be the inclusion of both quantitative traits (n = 96) and binary (disease) phenotypes (n = 123) in the human study [21] versus the use of only quantitative traits (n = 17) in the bovine study. For complex disease traits, a loss-of-function variant can induce a disease state. Consequently, such variants have a higher probability of being causal for complex disease phenotypes. Economic traits in dairy cattle are generally quantitative traits that are affected by genetic variants in many genes, each gene having a small effect. Furthermore, in addition to loss-of-function and gain-of-function mutations, most of the causal mutations that underlie economic traits are likely to belong to regulatory variants which control the up- or down-regulation of genes. Among the categories of enriched variants that we identified in our study, most of them had a link with the regulation of gene expression, such as cis-regulatory elements (e.g., upstream gene variants, downstream gene variants, and UTR) [43] and ncRNAs [40]. These variants can alter translation efficiency, particularly the synonymous variants [44], UTR [45], and ncRNAs [40]. In addition, they can affect transcript splicing, particularly the ncRNAs [40] and splice region variants. As a result, these variants can alter the functions of encoded proteins. A previous study demonstrated that regulatory elements are a major source of quantitative trait variation [46]. We go one step further and suggest that variants that affect both gene expression and protein translation (including translation efficiency and protein product stability) could be the source of quantitative trait variation.

Data from GWAS on stature in cattle were also used to check if an approach using an annotation category-based significance threshold can identify more associations than the use of a uniform Bonferroni multiple-testing correction threshold. A recent meta-analysis conducted in cattle [41] proposed a list of candidate genes that affect bovine stature. With the three annotation classification approaches that we used here, we identified two additional candidate genes, *TNNI2* and *TCP11*. Furthermore, comparison between the new list of genes reported here and that from a previous GWAS conducted on human height [42] revealed five additional genes with the four-category enrichment method, six additional genes with the five-category enrichment method, and ten additional genes with the eight-category enrichment method. Several reasons can explain why our approaches detected only a fraction of the candidate genes reported in previous studies [41, 42]: (1) loss of power for NKF and NKI categories, since we mixed variants with different probabilities due to lack of information for most variants; in this case, the enrichment estimation for NKF, NKI and unannotated variants that belong to another annotation category will be affected; (2) some of the causal variants reported in the meta-analysis study on bovine stature [41] and in the GWAS study on human height [42] do not segregate in the Nordic Holstein population; and (3) the relatively poor annotation of the bovine genome compared with the human genome. Nevertheless, our findings demonstrated that implementation of an annotation category-based significance threshold approach results in a larger number of high confidence candidate genes that include significant SNPs than the use of a flat Bonferroni multiple-testing significance threshold.

In this study, we observed distinct enrichments when two variant classification systems were used (e.g., 5 vs. 8 categories of classification) although the same number of potential causal variants was present in each system. There are two possible explanations for this observation: (1) the number of traits included in the analysis was small, thereby resulting in the detection of a limited number of QTL, e.g. there were only 19 high impact potential causal variants; with resampling, the number of high impact potential causal variants can fluctuate between extremes, as observed for the different replicates (see Additional file 3: Table S3); and (2) the number of annotation categories that were considered, and the underlying genetic architecture of the traits, may be contributing factors, e.g. a larger distribution of the enrichment level was observed across the categories established in this study when the variants were categorized into eight classes instead of five (see Additional file 3: Table S5).

In our study, we achieved slightly higher power with the classification of variants into categories based on annotations (Table 4 vs. Table 5 vs. Table 6). Further improvements are expected as the knowledge on the function of different genomic features in cattle increases. There are more and more studies that report the functions of non-coding sequences. For example, long ncRNAs have been identified as key regulators of chromatin states [47], microRNAs have been shown to play key roles in animal development and physiology [48], and *cis* regulatory elements may be located in 5′ or 3′ UTR. In combination with trans-regulatory elements, these elements regulate the level of gene transcription [49, 50]. In a human study [21], NKF variants were categorized according to the presence or absence of overlap with DHS. The group of variants that overlapped with DHS was more enriched with GWAS hits than the group of variants that did not overlap with DHS. These results provide support for future efforts to classify NKF variants, especially in cattle for which this information is not currently available. In our study, an ATAC-seq dataset [20] of accessible chromatin and a histone modification dataset (H3K27Ac and H3K4me3) [34] provided additional genomic information. In spite of these additional data, the OC variant category was only moderately enriched (Tables 2, 3) but these datasets were each generated from a single tissue from a few individuals, which could have introduced errors. Alternatively, predicted regulatory elements could be used, although they may introduce noise and reduce the estimated degree of enrichment. In our study, the NKF variants were divided into five categories according to their relation to UTR, OC, predicted RE [34], ncRNAs [35], and NKI variants. We anticipate that as the functional annotation of dairy cattle genomes improves, the power of this approach will increase.

## Conclusions

Analysis of the results from GWAS conducted on 17 quantitative traits in dairy cattle revealed high levels of enrichment for “high impact” variants, “moderate impact” variants, “low impact” variants, variants located in 3′ and 5′ UTR, and variants located in potential ncRNA regions. By setting category-based genome-wide significance thresholds based on these annotation enrichment data, we were able to identify new candidate genes that affect stature in cattle. We anticipate that future improvements in the annotation of the bovine genome, will optimize the usefulness of this approach even more.

## Additional files

**Additional file 1: Table S1.** Trait indices included in Nordic genetic evaluation analyzed for genome-wide association. For details on phenotypes and the model for estimation of breeding value, see http://www.nordicebv.info/. **Table S2.** Annotation categories.

**Additional file 2.** R code to estimate enrichment with resampling for further bootstrapping.

**Additional file 3: Tables S3.** Enrichment estimated in 100 bootstraps when variants are classified into five categories. **Tables S4.** Lead SNPs from genome-wide associated regions for stature in Nordic Holstein cattle. Base positions are given according to UMD 3.1.1. **Tables S5.** Enrichment estimated in 100 bootstraps when variants are classified into eight categories.

**Additional file 4: Figure S1.** Manhattan plot for association of SNPs with stature in Nordic Holstein cattle. Red horizontal line indicates the genome-wide significance level [− log10(P) = 8.5].

## Data Availability

Genome assembly data used in this study were obtained from the NCBI (ftp://ftp.ncbi.nlm.nih.gov/genomes/all/GCA/000/003/055/GCA_000003055.5_Bos_taurus_UMD_3.1.1). A subset of the whole-genome data from the 1000 Bull Genome Project is publicly available from dbSNP (http://www.ncbi.nlm.nih.gov/projects/SNP/) for variants and SRP039339 under PRJNA238491 for sequence data. For the remaining data, the Board of the 1000 Bull Genome Project Consortium should be contacted. All annotation information was obtained from a publicly available source (http://www.ensembl.org). Whole-genome sequences from Aarhus University and individual SNP genotype data are available only upon agreement with the breeding organization and should be requested directly from the authors.

## References

[1] Zimin AV, Delcher AL, Florea L, Kelley DR, Schatz MC, Puiu D (2009). A whole-genome assembly of the domestic cow, *Bos taurus*. Genome Biol.

[2] Daetwyler HD, Capitan A, Pausch H, Stothard P, van Binsbergen R, Brondum RF (2014). Whole-genome sequencing of 234 bulls facilitates mapping of monogenic and complex traits in cattle. Nat Genet.

[3] Hoglund JK, Sahana G, Guldbrandtsen B, Lund MS (2014). Validation of associations for female fertility traits in Nordic Holstein, Nordic Red and Jersey dairy cattle. BMC Genet.

[4] Kadri NK, Sahana G, Charlier C, Iso-Touru T, Guldbrandtsen B, Karim L (2014). A 660-Kb deletion with antagonistic effects on fertility and milk production segregates at high frequency in Nordic Red cattle: additional evidence for the common occurrence of balancing selection in livestock. PLoS Genet.

[5] Iso-Touru T, Sahana G, Guldbrandtsen B, Lund MS, Vilkki J (2016). Genome-wide association analysis of milk yield traits in Nordic Red Cattle using imputed whole genome sequence variants. BMC Genet.

[6] Cai Z, Guldbrandtsen B, Lund MS, Sahana G (2019). Dissecting closely linked association signals in combination with the mammalian phenotype database can identify candidate genes in dairy cattle. BMC Genet.

[7] Buitenhuis B, Janss LL, Poulsen NA, Larsen LB, Larsen MK, Sorensen P (2014). Genome-wide association and biological pathway analysis for milk-fat composition in Danish Holstein and Danish Jersey cattle. BMC Genomics.

[8] Sahana G, Lund MS, Andersson-Eklund L, Hastings N, Fernandez A, Iso-Touru T (2008). Fine-mapping QTL for mastitis resistance on BTA9 in three Nordic red cattle breeds. Anim Genet.

[9] Lund MS, Sahana G, Andersson-Eklund L, Hastings N, Fernandez A, Schulman N (2007). Joint analysis of quantitative trait loci for clinical mastitis and somatic cell score on five chromosomes in three Nordic dairy cattle breeds. J Dairy Sci.

[10] Cai Z, Guldbrandtsen B, Lund MS, Sahana G (2018). Prioritizing candidate genes post-GWAS using multiple sources of data for mastitis resistance in dairy cattle. BMC Genomics.

[11] Fang L, Sahana G, Su G, Yu Y, Zhang S, Lund MS (2017). Integrating sequence-based GWAS and RNA-Seq provides novel insights into the genetic basis of mastitis and milk production in dairy cattle. Sci Rep.

[12] Littlejohn MD, Tiplady K, Fink TA, Lehnert K, Lopdell T, Johnson T (2016). Sequence-based association analysis reveals an MGST1 eQTL with pleiotropic effects on bovine milk composition. Sci Rep.

[13] Zhu Z, Zhang F, Hu H, Bakshi A, Robinson MR, Powell JE (2016). Integration of summary data from GWAS and eQTL studies predicts complex trait gene targets. Nat Genet.

[14] Brown AA, Vinuela A, Delaneau O, Spector TD, Small KS, Dermitzakis ET (2017). Predicting causal variants affecting expression by using whole-genome sequencing and RNA-seq from multiple human tissues. Nat Genet.

[15] GTEx Consortium (2013). The genotype-tissue expression (GTEx) project. Nat Genet.

[16] Nishizaki SS, Boyle AP (2017). Mining the unknown: assigning function to noncoding single nucleotide polymorphisms. Trends Genet.

[17] McLaren W, Gil L, Hunt SE, Riat HS, Ritchie GR, Thormann A (2016). The ensembl variant effect predictor. Genome Biol.

[18] Pickrell JK (2014). Joint analysis of functional genomic data and genome-wide association studies of 18 human traits. Am J Hum Genet.

[19] Iversen ES, Lipton G, Clyde MA, Monteiro AN (2014). Functional annotation signatures of disease susceptibility loci improve SNP association analysis. BMC Genomics.

[20] Kichaev G, Yang WY, Lindstrom S, Hormozdiari F, Eskin E, Price AL (2014). Integrating functional data to prioritize causal variants in statistical fine-mapping studies. PLoS Genet.

[21] Sveinbjornsson G, Albrechtsen A, Zink F, Gudjonsson SA, Oddson A, Masson G (2016). Weighting sequence variants based on their annotation increases power of whole-genome association studies. Nat Genet.

[22] Goddard M (1985). A method of comparing sires evaluated in different countries. Livest Prod Sci.

[23] Schaeffer LR (1985). Model for international evaluation of dairy sires. Livest Prod Sci.

[24] Vuori K, Strandén I, Lidauer M, Mäntysaari E. MiX99-effective solver for large and complex linear mixed models. In: Proceedings of the 8th World congress on genetics applied to livestock production, 13–18 August 2006, Belo Horizonte; 2006. p. 27–33.

[25] Wu X, Guldbrandtsen B, Lund MS, Sahana G (2016). Association analysis for feet and legs disorders with whole-genome sequence variants in 3 dairy cattle breeds. J Dairy Sci.

[26] Brøndum RF, Rius-Vilarrasa E, Strandén I, Su G, Guldbrandtsen B, Fikse W (2011). Reliabilities of genomic prediction using combined reference data of the Nordic Red dairy cattle populations. J Dairy Sci.

[27] Howie B, Marchini J, Stephens M (2011). Genotype imputation with thousands of genomes. G3 (Bethesda).

[28] Fuchsberger C, Abecasis GR, Hinds DA (2015). minimac2: faster genotype imputation. Bioinformatics.

[29] Brondum RF, Guldbrandtsen B, Sahana G, Lund MS, Su G (2014). Strategies for imputation to whole genome sequence using a single or multi-breed reference population in cattle. BMC Genomics.

[30] Yang J, Lee SH, Goddard ME, Visscher PM (2011). GCTA: a tool for genome-wide complex trait analysis. Am J Hum Genet.

[31] Foissac S, Djebali S, Munyard K, Villa-Vialaneix N, Rau A, Muret K (2018). Livestock genome annotation: transcriptome and chromatin structure profiling in cattle, goat, chicken and pig. bioRxiv.

[32] Villar D, Berthelot C, Aldridge S, Rayner TF, Lukk M, Pignatelli M (2015). Enhancer evolution across 20 mammalian species. Cell.

[33] Hubbard T, Barker D, Birney E, Cameron G, Chen Y, Clark L (2002). The Ensembl genome database project. Nucleic Acids Res.

[34] Nguyen QH, Tellam RL, Naval-Sanchez M, Porto-Neto LR, Barendse W, Reverter A (2018). Mammalian genomic regulatory regions predicted by utilizing human genomics, transcriptomics, and epigenetics data. GigaScience.

[35] Petrov AI, Kay SJE, Gibson R, Kulesha E, Staines D, RNAcentral Consortium (2015). RNAcentral: an international database of ncRNA sequences. Nucleic Acids Res.

[36] Koudritsky M, Domany E (2008). Positional distribution of human transcription factor binding sites. Nucleic Acids Res.

[37] Yu CP, Lin JJ, Li WH (2016). Positional distribution of transcription factor binding sites in *Arabidopsis thaliana*. Sci Rep.

[38] Schork AJ, Thompson WK, Pham P, Torkamani A, Roddey JC, Sullivan PF (2013). All SNPs are not created equal: genome-wide association studies reveal a consistent pattern of enrichment among functionally annotated SNPs. PLoS Genet.

[39] Eddy SR (2001). Non-coding RNA genes and the modern RNA world. Nat Rev Genet.

[40] Mattick JS, Makunin IV (2006). Non-coding RNA. Hum Mol Genet.

[41] Bouwman AC, Daetwyler HD, Chamberlain AJ, Ponce CH, Sargolzaei M, Schenkel FS (2018). Meta-analysis of genome-wide association studies for cattle stature identifies common genes that regulate body size in mammals. Nat Genet.

[42] Marouli E, Graff M, Medina-Gomez C, Lo KS, Wood AR, Kjaer TR (2017). Rare and low-frequency coding variants alter human adult height. Nature.

[43] Wittkopp PJ, Kalay G (2012). Cis-regulatory elements: molecular mechanisms and evolutionary processes underlying divergence. Nat Rev Genet.

[44] Agashe D, Martinez-Gomez NC, Drummond DA, Marx CJ (2013). Good codons, bad transcript: large reductions in gene expression and fitness arising from synonymous mutations in a key enzyme. Mol Biol Evol.

[45] Wilkie GS, Dickson KS, Gray NK (2003). Regulation of mRNA translation by 5′- and 3′-UTR-binding factors. Trends Biochem Sci.

[46] Mackay TF (2004). The genetic architecture of quantitative traits: lessons from Drosophila. Curr Opin Genet Dev.

[47] Chu C, Qu K, Zhong FL, Artandi SE, Chang HY (2011). Genomic maps of long noncoding RNA occupancy reveal principles of RNA-chromatin interactions. Mol Cell.

[48] Ambros V (2004). The functions of animal microRNAs. Nature.

[49] Wittkopp PJ, Kalay G (2011). Cis-regulatory elements: molecular mechanisms and evolutionary processes underlying divergence. Nat Rev Genet.

[50] Yvert G, Brem RB, Whittle J, Akey JM, Foss E, Smith EN (2003). Trans-acting regulatory variation in Saccharomyces cerevisiae and the role of transcription factors. Nat Genet.

