# Supplementary material for: Weighting sequence variants based on their annotation increases the power of genome-wide association studies in dairy cattle
Source: Genet Sel Evol. 2019 May 10;51:20. doi: 10.1186/s12711-019-0463-9 (PMC6511139; doi:10.1186/s12711-019-0463-9)
Supplement: Supplementary file 1 — Additional file 1: Table S1. Trait indices included in Nordic genetic evaluation analyzed for genome-wide association. For details on phenotypes and the model for estimation of breeding value, see http://www.nordicebv.info/. Table S2. Annotation categories. [file 12711_2019_463_MOESM1_ESM.docx]

Weighting sequence variants based on their annotation increases power of genome-wide association studies in dairy cattle

Zexi Cai, Bernt Guldbrandtsen, Mogens Sandø Lund, Goutam Sahana

Additional file Table S1. Trait indices included in Nordic genetic evaluation analyzed for genome-wide association^1^. The descriptions are as in Nordic Cattle Genetic Evaluation homepage (http://www.nordicebv.info/http://www.nordicebv.info)

| Traits | Short description | Number of QTL | Observation |
| --- | --- | --- | --- |
| Milk/ Fat/ Protein | The estimated breeding values (EBVs) for milk production, protein and fat are based on production figures expressed in kilograms taken from milk records. The indices for protein and fat content are estimated from milk, protein and fat yield. | 26 / 27 / 34 | 5,034 / 5,034 / 5,034 |
| Yield index | Indexes for yield describe the genetic potential for milk, protein and fat production. The EBVs are combined over lactations. Standardized milk, fat and protein indexes are combined in the total yield index by means of weight factors based on economic calculations. | 36 | 5,043 |
| Growth | The index for growth describes the genetic growth ability of male offspring of bulls in terms of daily carcass gain and carcass conformation score. | 28 | 4,764 |
| Fertility | The index for fertility describes the genetic ability of the bulls’ daughters to start or resume breeding after calving, to show oestrus and to conceive at insemination. | 7 | 5,038 |
| Calving (direct)/ Calving (maternal) | The index for calving (maternal) and calving (direct) describes the genetic ability of calving ease and stillbirth. It is expressed by the daughters/ offspring of a bull. | 6 / 16 | 5,135 / 5,273 |
| Udder health | The index for udder health describes the genetic ability in resistance of the cow to mastitis. The breeding goal is to reduce the frequency of clinical mastitis. | 22 | 5,147 |
| General health | The index for general health describes the genetic resistance of bulls’ daughters to reproductive, digestive and feet and leg problems. | 6 | 4,916 |
| Feet and legs | Feet & Legs index describes genetic potential for feet and leg conformation. | 5 | 5,008 |
| Udder | Feet & Legs index describes genetic potential for feet and leg conformation; the shape and attachments of the udder. | 22 | 5,016 |
| Milkability | Indexes for milk ability describe how rapidly the cow can be milked. | 25 | 4,974 |
| Temperament | Indexes for temperament describe her general temperament. | 2 | 4,987 |
| Longevity | The index for longevity describes the genetic ability of the bulls’ daughters to survive. Bulls with high indices for longevity produce daughters with a longer productive life. | 11 | 4,672 |
| Body | The body index includes the type traits namely stature, body depth, chest width, dairy form, top line, rump width, rump angle. This index reflects closeness to “ideal” dairy type. | 8 | 4,972 |
| Nordic total merit index | All traits are combined into the Nordic Total Merit index (NTM). NTM describes the total economic potential determined by genetics. Cows with high NTM bring greater economic benefits to the farmer, and the use of sires with high NTM will give offspring of higher economic value. | NA | 2,272 |

^1^Details on phenotypes and model for estimation of breeding value, see <http://www.nordicebv.info/>

Note, milk and fat have one lead SNP overlap.

Table S2. The annotation category

| Classification I | Classification II | Classification III |  |
| --- | --- | --- | --- |
| (1) high impact variants (e.g., stop_gained, stop_lost, start_lost, frameshift, splice_acceptor, and splice_donor variants), | High impact (as in classification I) | High impact (as in classification I) | 1 |
| (2) moderate impact variants (missense_variants), | Moderate impact (as in classification I) | Moderate impact (as in classification I) | 2 |
| (3) low impact variants (synonymous, stop_retained, upstream_gene, downstream_gene and splice_region variants) | Low impact (as in classification I) | Low impact (as in classification I) | 3 |
| (4) other variants (including SNPs with a consequence predicted as “modifier ”). | (4.1) OC variants (annotated by VEP as “modifer” and located at ATAC-seq peaks or H3K27Ac and H3K4me3 peaks) | OC variants (as in classification II) | 4 |
|  | (4.2) variants with no known function i.e. NKF variants (including SNPs with a consequence predicted as “modifier”, which were not located in OC) | (4.2.1) variants located within 5’ and 3’ untranslated regions (UTR) | 5 |
|  |  | (4.2.2) variants located in predicted RE according to a recently proposed algorithm based on conservation among mammals | 6 |
|  |  | (4.2.3) variants located within ncRNAs retrieved from the RNAcentral database | 7 |
|  |  | (4.2.4) variants with no known information (NKI) predicted as “modifier” and not located in any of these four first three types of sequence | 8 |
