# Supplementary material for: Weighting sequence variants based on their annotation increases the power of genome-wide association studies in dairy cattle
Source: Genet Sel Evol. 2019 May 10;51:20. doi: 10.1186/s12711-019-0463-9 (PMC6511139; doi:10.1186/s12711-019-0463-9)
Supplement: Supplementary file 2 — Additional file 2. R code to estimate enrichment with resampling for further bootstrapping. [file 12711_2019_463_MOESM2_ESM.docx]

Weighting sequence variants based on their annotation increases power of genome-wide association studies in dairy cattle

Zexi Cai, Bernt Guldbrandtsen, Mogens Sandø Lund, Goutam Sahana

#################

# R script to illustrate how the MLE estimate can be calculated # Modified from Sveinbjornsson et al. Nature Genetics 48, 314-317 (2016) doi:10.1038/ng.3507

################

# Note that the data has to be preprocessed before this script can # be used. All LD calculations and

# definitions of association signals are e.g. part of the # preprocessing.

# The preprocessing can vary between datasets and we have therefore # not created a general software.

# The data includes one row for each SNP that is a part of an #association signal.

# AssocSignals identifies which association signal the SNP is in # SNPCHI is the chisq value of the SNP

# LEADCHI is the chisq of the most significant SNP in the #association signal

# Annot is the annotated functional categorie # SNPID is the ID of the SNP

#

#INPUT Data # head(dat)

# AssocSignal SNPID SNPCHI LEADCHI Annot

| # | 1 | 1 | 9.77505550 | 9.775055 | 1 |
| --- | --- | --- | --- | --- | --- |
| # | 1 | 2 | 0.05818727 | 9.775055 | 1 |
| # | 1 | 3 | 0.79595770 | 9.775055 | 5 |
| # | 1 | 4 | 0.60251661 | 9.775055 | 5 |
| # | 1 | 5 | 0.48252409 | 9.775055 | 5 |
| # | 1 | 6 | 0.07680607 | 9.775055 | 4 |
| # | 2 | 1 | 0.0276364585 | 8.744052 | 3 |
| # | 2 | 2 | 0.6687947495 | 8.744052 | 1 |
| # | 2 | 3 | 0.0706264871 | 8.744052 | 3 |
| # | 2 | 4 | 1.4361641588 | 8.744052 | 2 |
| # | 3 | 1 | 6.1434207676 | 6.143421 | 5 |
| # | 3 | 2 | 0.0011589648 | 6.143421 | 2 |
| # | 3 | 3 | 0.2952586988 | 6.143421 | 1 |

dat=read.table("enrich_5.txt_test",header=TRUE) require("optimx")

re_num = 100 #the total number of QTL resampling

signal_list = c(1:length(unique(dat$AssocSignal))) levels(dat$AssocSignal)=c(1:length(unique(dat$AssocSignal))) levels(dat$Annot)=c(1:length(unique(dat$Annot))) while(re_num) {

pick_signal = sample(signal_list,length(unique(dat

$AssocSignal)),replace=TRUE)

j=1 a=function(par){

par <- c(par, 1-sum(par)) #we want esimates to sum up to 1 if(sum(par<0)==0){ #negative estimates are not allowed

li=rep(0,length(levels(dat$AssocSignal))) for(i in pick_signal)

{

d=dat[dat$AssocSignal==i,] res=rep(0,length(d$SNPID)) for(m in c(1:length(d$SNPID)))

{

res[m]=as.numeric(exp((d$SNPCHI[m])-(d

$LEADCHI[m]))*prod(freq[d$Annot[-m]])*par[as.numeric(d$Annot[m])])

}

li[j]=sum(res) tmp_j = j + 1 j = tmp_j

}

li_log <- log(li) li_log[is.infinite(li_log)] <- NA return(sum(li_log,na.rm=T))

}

else return(-100000)

}

freq=c(1844/16503508,

51827/16503508,

7005/16503508,

889464/16503508,

15490368/16503508)

par=c(rep(0.2,4)) ## Give starting values for (1- #annot.classes); Try several starting values to ensure good #convergence

tryCatch({

result <- optimx(par,a ,method=c("Nelder- Mead"),control=list(fnscale=-1,maxit=5000))

tmp_num = re_num - 1 re_num = tmp_num

p=c(result$p1, result$p2, result$p3,result$p4) p=c(p, 1-sum(p))

enrich <- p/freq cat(enrich,"\n")

},

warning = function(war) { print(paste("warning: ",war))

},

error = function(err) { print(paste("error: ",err))

})

}

#The following are codes to calculate the weight for each annotation category and category-based Bonferroni correction threshold.

m <- c(1844,51827,70005,889464,15490368)

enrich <- c(4.915005,5.041424,405.0848,2.491081,0.721727) #

Enrichment for each class

w <- enrich/((1/sum(m))*(sum(m*enrich))) #Weights for each class

pb <- 0.05/sum(m) #Bonfarroni corrected threshold

pe <- pb*w #Threshold basedon enrichment

print(pe)
