# Supplementary material for: Weighting sequence variants based on their annotation increases the power of genome-wide association studies in dairy cattle
Source: Genet Sel Evol. 2019 May 10;51:20. doi: 10.1186/s12711-019-0463-9 (PMC6511139; doi:10.1186/s12711-019-0463-9)
Supplement: Supplementary file 4 — Additional file 4: Figure S1. Manhattan plot for association of SNPs with stature in Nordic Holstein cattle. Red horizontal line indicates the genome-wide significance level [− log10(P) = 8.5]. [file 12711_2019_463_MOESM4_ESM.docx]

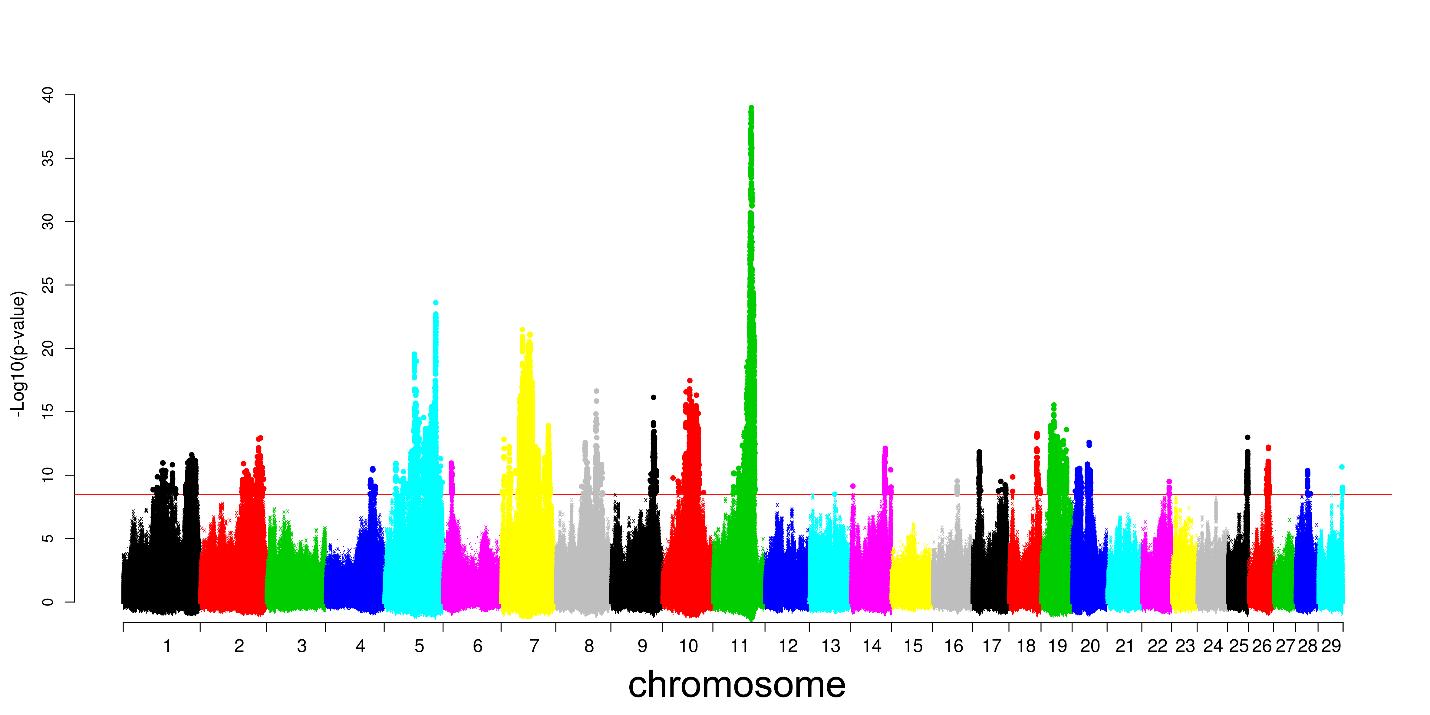


Figure S1. Manhattan plot for association of SNP with stature in Nordic Holstein cattle. Red horizontal line indicates genome-wide significance level [−log10(P) = 8.5]
